# Unhealthy Behaviours: An International Comparison

**DOI:** 10.1371/journal.pone.0141834

**Published:** 2015-10-29

**Authors:** Fabrizio Ferretti

**Affiliations:** Department of Communication and Economics, School of Social Sciences, University of Modena and Reggio Emilia, Reggio Emilia, Italy; The Scripps Research Institute Scripps Florida, UNITED STATES

## Abstract

In the current global economy, chronic non-communicable diseases (NCDs) have become the leading cause of death and a major health concern for both developed and developing countries. Among other factors, the worldwide spread of NCDs is driven by the globalisation of unhealthy habits. The purpose of this paper is to develop a simple statistic to measure, at the national level, the average population’s exposure to the main NCDs modifiable risk factors. The approach and methodology followed by the United Nations Development Programme to compute the Human Development Index (*HDI*) is applied to four basic indicators of NCD-related preventable risk factors (alcohol consumption, excess caloric intake, non-balanced diet and tobacco use) in 112 countries worldwide in 2012–14. We obtain a summary composite index, which we call the Unhealthy Behaviour Index (*UBI*), which ranks countries by the average level of the unhealthy habits (drinking, eating and smoking) of their populations. We find that Belarus and Russian federation are the two countries with the unhealthiest NCD-related lifestyle. With the exception of Canada, the first twenty populations more exposed to the main NCDs preventable risk factors all live in European countries, and mainly in countries of Eastern Europe. Overall, the *UBI* tends to increase along with the level of human development. In medium, high and very high *HDI* countries, however, the same level of human development may be associated with very different kinds of NCD-related lifestyles. Finally, economic growth may push populations toward either more unhealthy or healthy habits, depending on the countries’ level of development; the elasticity of unhealthy habits with respect to income per capita is positive (but less than one: on average 0.6) until $30,000, decreases as income rises, and becomes negative (around -0.3) in very high income countries.

## Introduction

Once considered ‘diseases of affluence’ that affected mostly elderly and wealthy people in Western advanced countries, today, non-communicable diseases (NCDs) have become one of the major global health concerns, as well as the leading cause of death worldwide [1]. According to the latest World Health Organization (WHO) global report, NCDs: “…were responsible for 38 million (68%) of the world’s 56 million deaths in 2012. More than 40% of them (16 million) were premature deaths under the age of 70 years. Almost three-quarters of all NCD deaths (28 million) and the majority of premature deaths (82%) occur in low- and middle-income countries” ([2], p. XI).

The increasing worldwide burden of NCDs is the result of complicated interactions between several demographic, economic and social structural changes [3], and is strongly associated with the globalisation of unhealthy lifestyles [4–6]. The leading four chronic NCDs—that is, cardiovascular diseases, chronic respiratory diseases, cancers, and diabetes [7]—are characterised by a complex aetiology, but generally stem from a combination of non-modifiable risk factors (e.g., sex, age and the inborn genetic characteristics of individuals), and a well-known set of modifiable risk factors: primarily, tobacco use, alcohol abuse, qualitative and quantitative unhealthy nutrition, lack of physical activity, environmental pollution and chronic infection [8]. This is why epidemiological studies emphasise the role of primary prevention to tackle NCDs and suggest effective ways to drastically reduce the global incidence of NCDs by controlling the main lifestyle-related risk factors in each country [9,10].

Recent developments in NCD epidemiology highlight the importance of measuring the combined effects of multiple lifestyle risk behaviours on people’s health outcomes [11]. To date, the research has helped to understand and explain this phenomenon at the individual (i.e., microeconomic) level [12]. The purpose of this paper is to develop a simple statistic in order to measure the average population’s exposure to the main NCD modifiable risk factors at the country (i.e., macroeconomic) level. In what follows, the approach and methodology followed by the United Nations Development Programme (UNDP) to compute the *HDI* (Human Development Index) [13] is applied to four of the main preventable risk factors that underlie the leading NCDs (alcohol abuse, excess caloric intake, non-balanced diet, and tobacco use) in 112 countries worldwide for the period 2012–14. The result is a summary composite index—that will be called the ‘Unhealthy Behaviour Index (*UBI*)’—which allows us to rank countries by the average level of the unhealthy (drinking, eating and smoking) habits of their respective populations.

## Methods and Data

**Fig 1:**
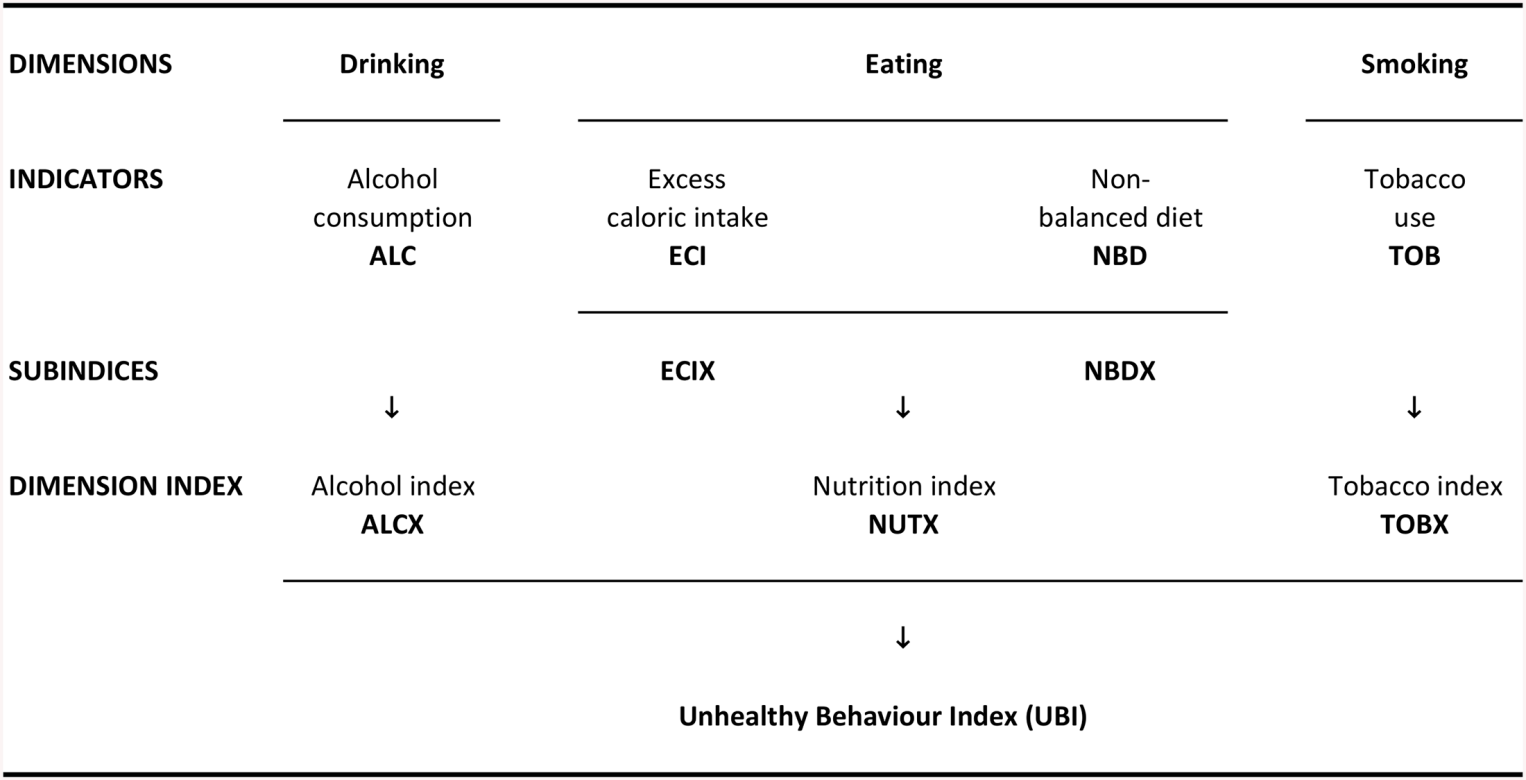
The Unhealthy Behaviour Index (*UBI*): Graphical presentation.

It is often useful to construct a composite indicator to summarise a wide range of indicators of a multi-dimensional phenomenon in a single statistic [14]. In this paper, we construct the *UBI*, a summary measure of three fundamental dimensions of people’s health-related habits and behaviours: drinking, eating and smoking (Fig 1). These key lifestyle factors are captured here by four basic indicators of the main NCD modifiable risk factors [15]: i.e. alcohol consumption (*ALC*), excess caloric intake (*ECI*), non-balanced diet (that is, a diet too rich in total fat and protein, *NBD*) and tobacco use (*TOB*).

Each indicator is transformed into a corresponding normalised index (*I*_*x*_) according to the standard *HDI* methodology [16] as follows:

$$I_{x}\;=\;\frac{act_{x}\;-\;{\text{min}}_{x}}{{\text{max}}_{x}\;-\;{\text{min}}_{x}}$$ (1)

where *act*_*x*_, *min*_*x*_ and *max*_*x*_ stand for the actual, minimum and maximum value of the underlying indicator. For the eating dimension, Eq 1 is applied to each of the two subcomponents (*ECI* and *NBD*). Then, a geometric mean of the resulting sub-indices (*ECIX* and *NBDX*) is created, and Eq 1 is applied again to the geometric mean of these two indices in order to obtain the nutrition dimension index (*NUTX*). Finally, *UBI* is computed as the geometric mean of the three normalised dimension indices—that is, alcohol (*ALCX*), nutrition (*NUTX*), and tobacco (*TOBX*)—as follows:

UBI = (ALCX13×NUTX13×TOBX13) (2)

Computing Eq 2 requires data on several variables (Table 1). Specifically, *ALC* is measured by the per capita consumption of pure alcohol (litres/person15+/year) estimated for 2012 by the WHO [2,17]. *TOB* is measured by the per capita consumption of cigarettes (number/person/year) estimated for 2012 by Ng et al. [18]. *ECI* and *NBD* are measured by the per capita intake of energy (kcal/day) and of total fat and protein (g/day) over the recommended level, estimated for 2012–14 (three-year average) by the United Nations Food and Agricultural Organization (FAO) and the WHO [19,20].

**Table 1:** Variables and goalposts for the Unhealthy Behaviour Index.

Variable	Code	Description	Source	Obs. max.	Min.
Alcohol consumption	***ALC***	Per capita consumption of pure alcohol (litres/person 15+/year)^a^	WHO (2014)	17.8 (Belarus, 2012)	0
Excess caloric intake	***ECI***	Actual—Recommended caloric intake ECI = DES—ADER (kcal/person/day)^c^	FAO (2014)	1,349.5 (Turkey, 2012–2014)^b^	0
Non-balanced diet	***NBD***	Actual—Recommended fat and protein intake NBD = AFPS—RFPI (g/person/day)^d^	FAO (2014) and FAO/WHO (2003)	145.1 (Israel, 2012–2014)	0
Tobacco use	***TOB***	Per capita cigarettes consumption (number/person/year)	Ng et al. (2012)	3,385 (Cyprus, 2012)	0

^a^15+ = person aged 15 or older.

^b^2012–2014 = three years average.

^c^DES = Dietary Energy Supply; ADER = Average Dietary Energy Requirement.

^d^AFPS = Average fat and protein supply; RFPI = Recommended fat and protein intake.

About the two *NUTX* sub-indices, *ECI* is calculated from the FAO database [19] as the difference between the Dietary Energy Supply (*DES*) and the Average Dietary Energy Requirement (*ADER*), whereas *NBD* is calculated, again, from the FAO database as the difference between the actual and the recommended total fat and protein intake (*AFPS* and *RFPI*, respectively). Intake recommendations are taken from the joint FAO-WHO intake goals to prevent diet- and nutrition-related chronic NCDs [20] (see S1 Appendix for details on these calculations). *ECI* and *NBD* are, therefore, basic indicators of a population’s incorrect eating habits, characterised by the excessive and unhealthy consumption of calorie-, fat-, and protein-dense foods.

Minimum and maximum values (or goalposts) for each indicator are set in order to transform variables into a corresponding index between 0 and 1. The main purpose of the *UBI* is to provide a country summary measure of multiple health risk behaviours, in which populations with the most NCD-related unhealthy habits receive the highest *UBI* scores. To this end, maximum values are simply the highest observed values in the sample—i.e. *ALC*, 17.8 litres (Belarus), *ECI*, 1,349.5 kcal (Turkey), *NBD*, 145.1 g (Israel), and *TOB*, 3,385 cigarettes (Cyprus), respectively. Minimum values are set equal zero for all indicators.

The choice to set *min*_*x*_ equal to zero is straightforward for tobacco use, as the optimal level of cigarette consumption is always equal to zero. In a similar way, since positive values of both *ECI* and *NBD* measure excess dietary deviations from the international standard guidelines to prevent NCDs, it is reasonable to set the minimum deviation from the correct energy, total fat, and protein intake equal to zero. Finally, despite moderate alcohol consumption seeming to provide mild protection against selected NCDs [21], the minimum value of *ALC* is also set equal to zero, because several studies suggest that this moderate level is very low [22–24], and is associated not with alcohol itself, but to the polyphenols contained only in select alcoholic beverages (especially red wine) [25].

Combining the WHO [2,17], Ng et al. [18] and FAO [19] databases, there are 159 countries with no missing data for the four indicators (*ALC*, *ECI*, *NBD* and *TOB*). However, 47 countries—which have a history of issues of undernourishment—have negative values for either or both *ECI* and *NBD*. Since our focus is on people’s unhealthy lifestyle choices, we will compute the index only within the subset of the 112 remaining countries, where both nutrition indicators assume positive values (see Tables B and C in S1 File for the full and the reduced database).

Let us compute, as an example, the *UBI* in the United Kingdom (Table 2). In 2012, the estimated UK consumption of alcohol and cigarettes per capita was 11.4 litres/year and 998 cigarettes/year, respectively. Furthermore, from 2012–14, the UK per capita intake of calories and of total fat and protein were estimated well above the requirement of the population to prevent NCDs (specifically, an excess of 922.3 kcal/day and 106.8 g/day, respectively). Given the goalposts, according to Eq 1, these figures give:

ALCX UK= 11.4 - 017.8 - 0 = 0.640 (3)

TOBX UK= 998 - 03,385.0 - 0 = 0.295 (4)

ECIX UK= 922.3 - 01,349.5 - 0 = 0.683 (5)

NBDX UK= 106.8 - 0145.1 - 0 = 0.736 (6)

Therefore, computing the geometric mean of *ECIX*_*UK*_ and *NBDX*_*UK*_, and reapplying Eq 1 to the result, gives:

NUTX UK= 0.683×0.736 - 00.967 - 0 = 0.734 (7)

This value entered into Eq 2, along with *ALCX*_*UK*_ and *TOBX*_*UK*_ gives:

UBI UK= 0.640×0.734×0.2953 = 0.517 (8)

Equation shows a *UBI* value for the UK’s population of around 0.52, in which a diet too rich in both fat and protein, an excess of caloric intake, and a non-negligible level of alcohol consumption are partially offset by a relatively modest tobacco use.

**Table 2:** Calculating the Unhealthy Behaviour Index (example: United Kingdom).

Variable	Code	United Kingdom	Max	Min	Subindices		Dimension index
Alcohol consumption	*ALC*	11.4	17.8	0	*ALCX*		**0.640**
Excess caloric intake	*ECI*	922.3	1,349.5	0	*ECIX*	0.683	
Non balanced diet	*NBD*	106.8	145.1	0	*NBDX*	0.736	
					gm (*ECIX*, *NBDX*)^a^	0.709	
					*NUTX*		**0.734**
Tobacco consumption	*TOB*	998.0	3,385	0	*TOBX*		**0.295**
					**Unhealthy Behaviour Index—*UBI***		**0.517**

^a^gm (ECIX, NBDX) = geometric mean of ECIX and NBDX.

## Results

The results of applying the above methodology to the set of 112 countries worldwide are summarised in Table 3, where the first 20 countries for the unhealthy habits of their populations are listed, as measured by the *UBI* and by its three dimension indices (see Tables C and D in S1 File for the full results and the full country ranking).

**Table 3:** Ranking of the first ten countries by *UBI* and dimension indices.

	Country	*UBI*	Country	*ALCX*	Country	*NUTX*	Country	*TOBX*
**1**	Belarus	0.773	Belarus	1.000	Israel	1.000	Cyprus	1.000
**2**	Russian Fed.	0.694	Lithuania	0.949	Austria	0.954	Malta	0.961
**3**	Greece	0.687	Moldova	0.904	United States	0.919	Belarus	0.856
**4**	Czech Rep.	0.668	Russian Fed.	0.831	Belgium	0.917	Russian Fed.	0.838
**5**	Ireland	0.651	Czech Rep.	0.787	France	0.874	Croatia	0.819
**6**	Montenegro	0.647	Ukraine	0.787	Canada	0.837	Moldova	0.816
**7**	Austria	0.644	Romania	0.742	Luxembourg	0.827	Lebanon	0.804
**8**	France	0.634	Croatia	0.730	Montenegro	0.822	Greece	0.803
**9**	Romania	0.633	Slovakia	0.702	Italy	0.821	St. Vincent^a^	0.795
**10**	Croatia	0.630	Hungary	0.697	Ireland	0.800	Bosnia-Herz.	0.788
**11**	Lithuania	0.626	France	0.691	Greece	0.780	Macedonia	0.748
**12**	Malta	0.624	Serbia	0.691	Norway	0.776	Slovakia	0.716
**13**	Luxembourg	0.615	Portugal	0.685	Iceland	0.775	Montenegro	0.673
**14**	Belgium	0.606	Latvia	0.674	Turkey	0.772	Serbia	0.672
**15**	Switzerland	0.599	Australia	0.669	Germany	0.751	Armenia	0.664
**16**	Canada	0.590	Luxembourg	0.669	Portugal	0.746	Estonia	0.661
**17**	Poland	0.589	Finland	0.657	Switzerland	0.738	Korea Rep.	0.646
**18**	Latvia	0.584	Poland	0.652	United Kingdom	0.734	Czech Rep.	0.625
**19**	Slovenia	0.579	Germany	0.646	Australia	0.702	Uruguay	0.610
**20**	Kazakhstan	0.574	United Kingdom	0.640	Lithuania	0.695	Ireland	0.608

^a^ Saint Vincent and the Grenadines

Belarus is the population with the highest *UBI* value in the sample (0.77), and thus, with the worst exposure to NCD lifestyle risk factors, strictly followed by the populations of the Russian Federation and Greece (0.69 and 0.68, respectively). It is worthwhile to note that, with the exception of Canada, the first column of Table 3 contains only European countries: mainly countries from Eastern Europe. This evidence is even more striking for the drinking dimension, where Australia is the only non-European country, and the first top ten positions are all occupied by Eastern European populations. In the same way, an NCD prone lifestyle for tobacco use tends to prevail in countries in Eastern Europe (especially from Balkans) and from the Mediterranean region (i.e., Cyprus, Greece, and Malta). Finally, the composition of the first ten populations for unhealthy habits changes partially for the eating dimension. Israel has the highest score, and Eastern European countries are replaced by European Union (Austria, Belgium, France, Luxemburg, Italy and Ireland) and North American (the USA and Canada) countries.

**Fig 2:**
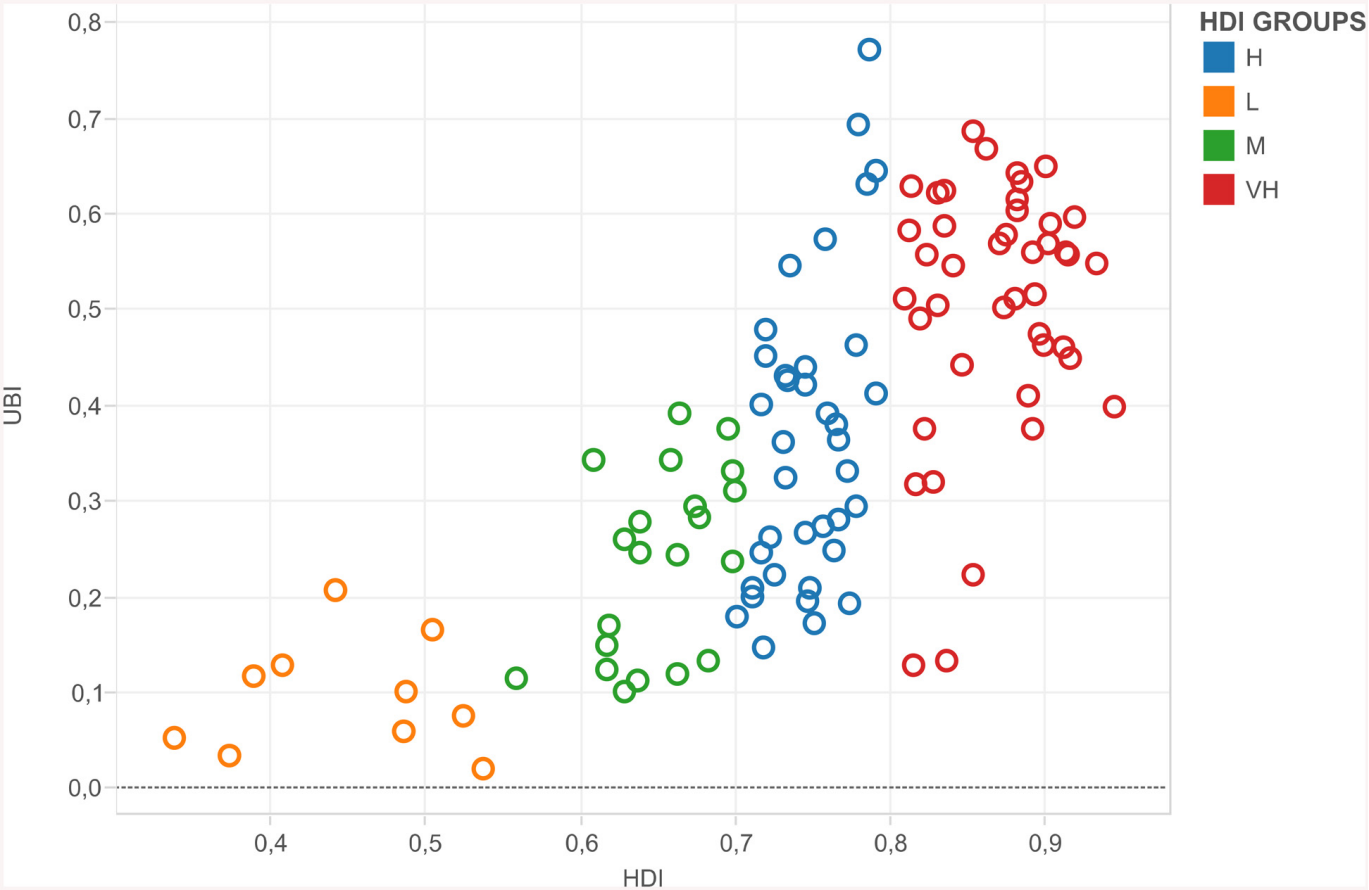
The Unhealthy Behaviour Index and the Human Development Index.

Overall, unhealthy lifestyles tend to increase with the level of human development, as measured by the *HDI*. This is shown in Fig 2, which plots the *UBI* against the *HDI* for each country in the sample. There is a clear positive relationship between the average levels of human development and unhealthy habits. However, by classifying countries according to their *HDI* into the four main UNDP’s groups (i.e., very high, high, medium and low *HDI*) [26], it is interesting to note that, in medium, and especially in high and very high *HDI* countries, the relationship between *UBI* and *HDI* tends to become nearly vertical. In other words, the same level of human development may be associated with very different kinds of NCD-related lifestyles.

A fundamental component of the *HDI* is the income per capita (*Y*), usually measured by the Gross National Income per person (*GNIpc*, in PPP $ [26]). Table 4 shows the average value of the *UBI* and its components when countries are classified according to their per capita income level. On average, the *UBI* more than triples in ‘rich’ countries (i.e., with a *GNIpc* greater than $30,000) with respect to the poorest one (where the *GNIpc* is less than $5,000): 0.48 and 0.15, respectively. However, the upper-middle (i.e., with a *GNIpc* between $15,001 and $30,000) and high-income countries show around the same unhealthy outcome, with better performance of the latter for the drinking and, especially, smoking dimensions. On the contrary, the excess of both energy and total fat-protein intake always increases from low to high income countries.

**Table 4:** Unhealthy Behaviour Index and components by income groups.

Income groups	*UBI*	η_*UY*_ ^a^	*ALC*	*ECI*	*NBD*	*TOB*	*n* ^b^
High (GNI pc^c^ > 30.000)	0.488	-0.29	8.5	879.7	96.9	1,487	26
Upper middle (GNI pc 15.001–30.000)	0.476	0.52	9.3	720.3	58.1	1,833	33
Lower middle (GNI pc 5.001–15.000)	0.292	0.66	6.4	564.1	27.7	1,286	37
Low (GNI pc < or = 5.000)	0.153	0.71	2.9	483.1	20.3	742	16

^a^η_*UY*_ = income elasticity of the UBI

^b^ *n* = number of countries of the sample in the income groups.

^c^ GNI pc = Gross National Income per capita 2013 (2011, PPP $) (UNDP, 2014), PPP is purchasing power parity.

The relationship between economic development and unhealthy habits can be further examined by computing the elasticity of the *UBI* with respect to income per capita—i.e. η_*UY*_ = (*dUBI*/*dY*) × (*Y*/*UBI*)—which measures the percentage change of the *UBI* when income changes by 1 percent. The result of the computation may help to understand and measure the impact of economic growth on health-related lifestyles. To do this, we regress *GNIpc* on *UBI* with a simple quadratic model (*UBI = β*_0_ + *β*_1_ *GNIpc* + *β*_2_ *GNIpc* ^2^ + ε) that, estimated using White's coefficient covariance matrix to obtain heteroskedasticity robust standard errors, gives:

$$\begin{array}{lll} UBI\;=0.10 & +\text{ 2.25E}-05GNIpc & \text{- 2.78E}-10GNIpc^{2}\\ & \text{(1.88E}-06) & \text{(3.00E}-11)\\ & t\;=11.92 & -9.28 \end{array}$$ (9)

(*n* = 112, Adj. *R-Sq*. = 0.53). Therefore, η_*UY*_ is computed from Eq 9 as (*β*_1_ + 2*β*_2_ *GNIpc*) × (*GNIpc*/*UBI*), by using the average value of *UBI* and *GNIpc* in each income group. The results, as shown in column 3 of Table 4, indicate that health-related habits are relatively inelastic to income changes (all coefficients are less than one). But, it is noteworthy that η_*UY*_ is positive (on average, 0.6) for populations with per capita income less than $30,000, decreases from 0.7 to 0.5 as income rises from $5,000 to $30,000 per capita, and becomes negative (on average, -0.3) in high income countries. In other words, sustained increases in income per capita may push populations toward more either unhealthy or healthy habits. However, both the direction and magnitude of these changes depend on the country’s level of economic development. Finally, by setting the first derivative of Eq 9 equal to zero and solving for *GNIpc*, we can find an income turning point of about $40,500. This is the threshold income level beyond which η_*UY*_ changes its sign, and economic growth starts exerting a positive effect on people’s NCD-related lifestyles.

## Discussion

Within a simple model of consumer behaviour, these empirical findings may help to better understand the channels through which economic growth and human development affect the incidence of NCDs in both advanced and emerging countries. Thus, in this section, we present an economic framework to ground our empirical work and discuss its implications for the epidemiology of NCDs.

In order to capture people’s exposure to lifestyle risk factors, one may consider an NCD-related consumption bundle in which each item is described by the list of its healthy and unhealthy characteristics. The whole set of these characteristics determines a more or a less risk prone lifestyle. To simplify, let us consider a representative market basket composed of healthy goods (*h*, such as fruits and vegetables) and unhealthy goods (*u*, such as high-fat and calorie-dense foods) [27].

Both kinds of goods yield immediate satisfaction, but exert opposite effects on future health outcomes. A main feature of NCD epidemiology is the delay between illness onset and the exposure to risk factors. That is, today’s incidence rate is affected by yesterday’s exposure, and today’s exposure will affect tomorrow’s incidence rate. Consuming *h* and *u* increases today’s utility. However, utility from *u* comes at a price of a greater exposure to NCD risk factors, and thus, of a greater likelihood to develop one or more NCDs in the future (and vice versa for *h*, whose consumption gives utility today and protects from NCDs, providing future health benefits) [28].

**Fig 3:**
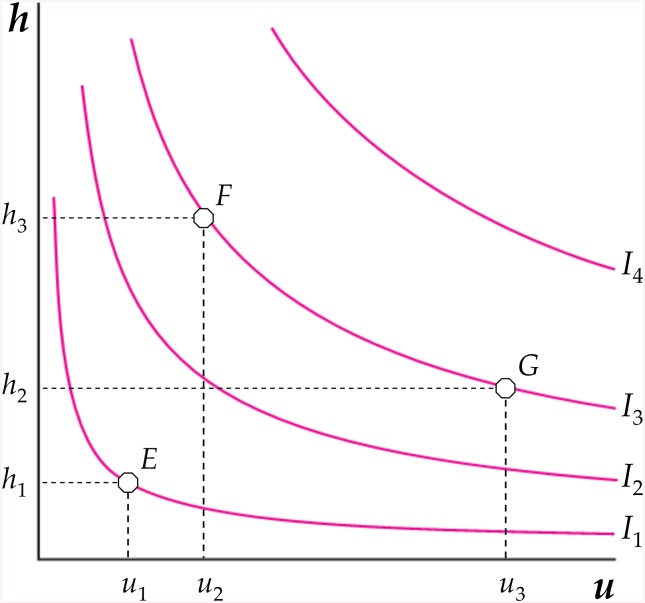
Indifference curves for *u* and *h*.

At any given time, the individual’s preferences for *h* and *u* can be usefully described by an indifference map (as shown in Fig 3). Each indifference curve (*I*_1_, *I*_2_, *I*_3_ and *I*_4_) represents all combinations of *h* and *u* that yields the same level of ‘today’s utility’ (i.e., satisfaction in the current period). Because both more *h* and *u* make the individual better off today, indifference curves are downward sloping, and the further the curves are from the origin, the greater is the level of satisfaction that they represent [27].

At a low level of utility, however, indifference curves (such as *I*_1_) are nearly right angles; that is, people are ‘too poor’ to be able to choose between *h* and *u*. In these contexts, where a consumption bundle, such as *E*, is the one that is affordable to the majority of the population, analysing unhealthy behaviours is somewhat inappropriate. This is why the *UBI* is computed by deleting all the poorest countries from the sample. In other words, our analysis applies to societies with medium, high or very high levels of human development. Indeed, in each subset of these countries (as clearly shown in Fig 2), the same level of human development is associated with very different *UBI* values. This means that, moving away from the origin in Fig 3, indifference curves become flatter (such as *I*_2_ or *I*_3_), and people may exert some degree of freedom on their health-related habits. At these corresponding level of utility, consumers are relatively free to choose between different consumption bundles (such as *F* or *G*, on *I*_3_), all of which represent a more healthy or unhealthy lifestyle, respectively.

**Fig 4:**
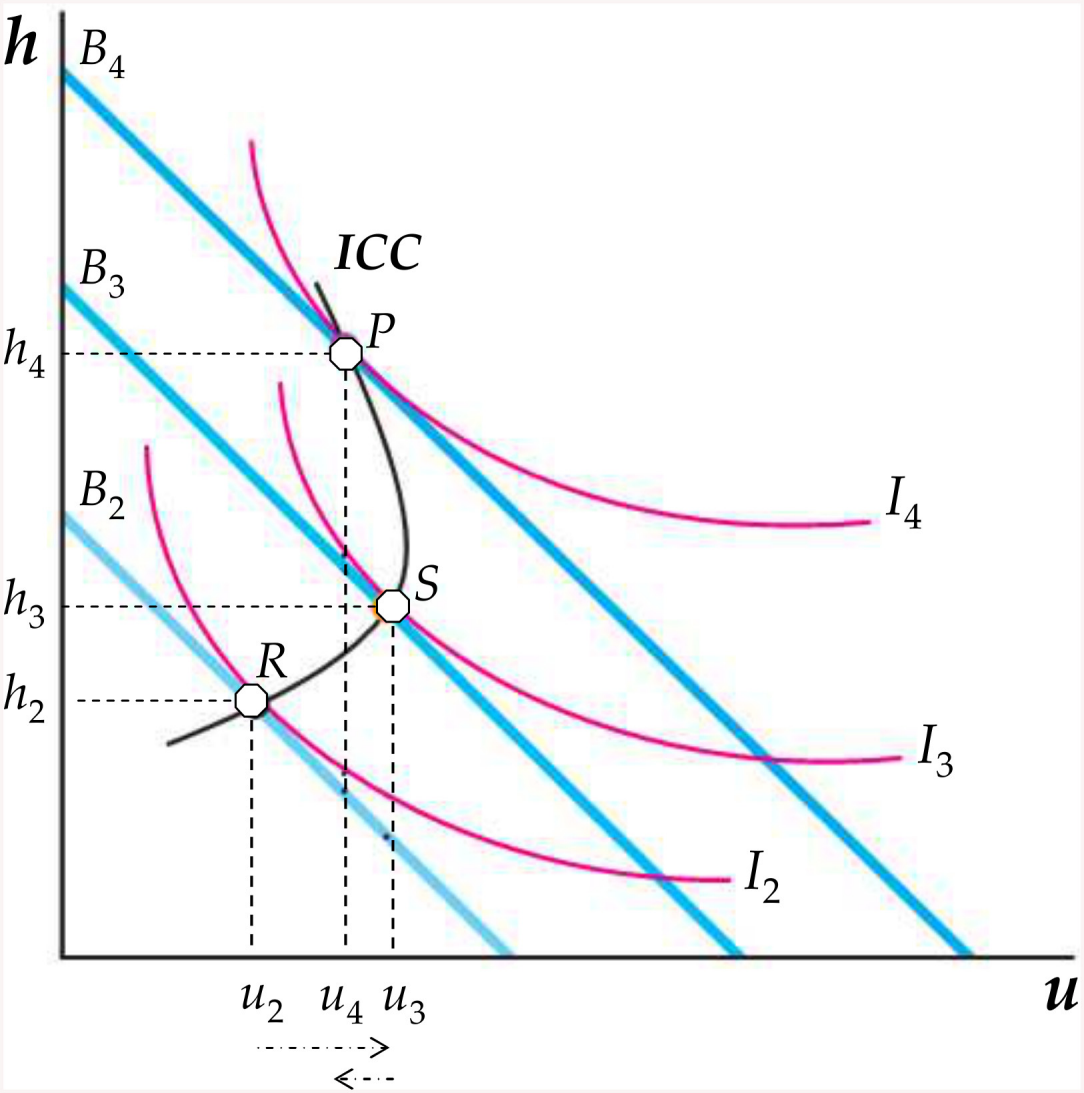
The income consumption curve for *u* and *h*.

In this simplified model, people’s exposure to NCD lifestyle risk factors depends on consumption choices of *h* and *u*. These kinds of choices, in turn, depend not only on preferences, but also on budget constraints, i.e. on average income (*Y*) and goods prices (*p*_*h*_ and *p*_*u*_). This is shown in Fig 4, where each straight line (*B*_2_, *B*_3_ and *B*_4_) indicates which consumption bundles are affordable at different income levels. If *Y* = *Y*_2_, for example, *B*_2_ shows all feasible quantities of *h* and *u* for which—given *p*_*h*_ and *p*_*u*_—total expenditure equals a consumer’s income (i.e., *p*_*h*_ *h* + *p*_*u*_ *u* = *Y*_2_ or, rearranging, *h* = (*Y*_2_/*p*_*h*_)—(*p*_*u*_/*p*_*h*_)×*u*).

With a limited purchasing power, the consumer’s optimal bundle is the result of a constrained optimisation problem: choosing the combination of *h* and *u* that yields the highest utility, given the restrictions imposed by income and prices. For a given constraint, such as *B*_2_ in Fig 4, the optimal bundle (*h*_2_, *u*_2_)—and thus, the more or less risk prone lifestyle—occurs at the tangency between the budget line and the highest (i.e., further from the origin) indifference curve (point *R*).

The diminishing value of η_UY_, reported in Table 4, finds a straightforward explanation in Fig 4. Consumption patterns are characterised by a hierarchical structure, determined by the interplay between biological, economic and social factors. Therefore, the demand for goods, such as *h* and *u*, does not expand proportionally with economic growth and human development. Above threshold levels of disposable income (such as *Y*_3_ or *Y*_4_, in Fig 4), people start following new lifestyles, modifying accordingly their consumption patterns [29,30]. Specifically, as income increases (for example, from *Y*_2_ to *Y*_4_), the budget constraint shifts outward (from *B*_2_ to *B*_4_). Consumers can reach indifference curves further from the origin and choose better (i.e., higher utility) consumption bundles such as (*h*_3_, *u*_3_) or (*h*_4_, *u*_4_).

The path connecting the utility-maximising bundles (points *R*, *S*, *P* in Fig 4) shows how consumers change their health-related behaviours as income increases, ceteris paribus. This income-consumption curve (*ICC*) becomes steeper at higher levels of utility, showing that a decreasing proportion of income is spent on *u* (the demand for *u* increases, but less than income). According to our results, this is the case in low, middle and upper-middle income countries, where η_UY_ is less than one, and tends to diminish as income rises. Conversely, the backward bending portion of *ICC* above point *S* indicates a shifting toward more healthy lifestyles. This case applies to high income countries, where η_UY_ is negative; hence, *u* become an inferior good (that is, a good for which consumption decreases when income rises).

These findings may have several interesting implications for public health. The income elasticity of health outcomes (η_*HY*_) is a frequently used metric to summarise the impact of economic growth on people’s health [31]. Specifically, η_*HY*_ is the percentage change in a given measure of a population’s health status (*H*) divided by the percentage change in income per capita (*Y*). That is, (Δ*H*/*H*)/(Δ*Y*/*Y*), or more usefully:

η HY=  dHdY×YH (10)

which gives the percentage change in health outcomes resulting from a 1% change in average income. Now, let us denote with *H* the age-standardised incidence rate of the leading NCDs. In any population, given the inborn characteristics of individuals (*Z*), *H* depends on at least two fundamental factors: health care (*HC*) and health-related behaviours (*HB*) [32]. This relationship between health inputs and outcome may be described by a ‘health production function’ [33] as follows:

H = f(HC, HB, Z) (11)

where *HC* is a measure of the resources devoted to preventing NCDs, and *HB* is a summary measure of people’s exposure to NCD lifestyle risk factors.

In this paper, we focus on *HB*. For our purposes, both *Z* and *HC* can be treated as exogenous variables of the model, whose value is taken as given. Thus, we can rewrite Eq 11 as a relation between incidence rate and health-related behaviours—i.e. *H* = *f*(*HB*)—holding all other factors affecting *H* constant. Moreover, we are able to replace the generic *HB* variable with the *UBI*, and make the dependence of health-related lifestyles from income per capita explicit: *UBI* = *g*(*Y*). As a result, Eq 11 becomes:

H = f[g(Y)] (12)

This expression simply states that changes in income lead to changes in health-related habits; in turn, changes in health-related habits affect a population’s exposure to lifestyle risk factors, and thus, lead to changes in health outcomes.

Finally, in order to measure the magnitudes of these changes [34], let us differentiate *H* with respect to *Y* in Eq 12. By applying the chain rule, this gives:

$$\frac{dH}{dY}\;=\;\frac{dH}{dUBI}\times \frac{dUBI}{dY}$$ (13)

Remembering (from Eq 10) that *η*_*HY*_ is *dH*/*dY* multiplied by the income-health ratio (*Y*/*H*), and since *Y*/*H* can also be written as (*Y*/*UBI*) × (*UBI*/*Y*), after some manipulations, the expression for *η*_*HY*_ becomes:

η HY=  dHdUBIUBIH×dUBIdYYUBI (14)

In Eq 14, the ‘reactivity’ of the NCD incidence rate to economic growth results from the product of two key factors: the elasticity of a population’s health status with respect to health-related lifestyles (η_*HU*_) multiplied by the elasticity of a population’s health-related lifestyles with respect to income per capita (that is, by the income elasticity of the *UBI*, η_*UY*_):

η HY=  η×HU ηUY (15)

It is worth noting that η_*HU*_ and η_*UY*_ are output and income elasticity, respectively. On the one hand, η_*HU*_ tells us how much *H* responds to a 1% change in the *UBI*. Its value is, therefore, largely determined by the technical and biological constraints that characterise the health production process. On the other hand, η_*UY*_ tells us how much the *UBI* responds to a 1% change in *Y*. Its value depends mainly on people’s preferences as consumers and citizens about health-related lifestyle choices; thus, it could be properly manipulated by public health prevention programmes.

As a result, to understand and predict, at a macroeconomic level, how *H* may evolve in a growing economy—given *Z* and *HC*–it is crucial to measure not only η_*HU*_, but also η_*UY*_, which gives both the direction and magnitude of a population’s changes in health-related lifestyles in response to better living standards. In light of Eq 15, our results suggest that, other things being equal, up to a per capita income of about $40,500 (i.e., an extremely high income level), economic growth tends to increase unhealthy habits. Therefore, albeit decreasing and less than one, the positive value of η_*UY*_ for a large majority of countries strongly confirms the role of primordial and primary prevention to reduce the incidence of NCDs throughout the world.

## Conclusions

The main purpose of this study was to describe a straightforward method for calculating, at a macroeconomic level, an index of NCD-related unhealthy behaviours. Despite its highly simplified nature, the *UBI* creates a meaningful country ranking and offers some insights into the relation between economic development and the incidence rate of the main NCDs. Our results suggest that a significant part of the world’s population still lives in regions where economic growth tends to push people toward ‘Westernised’, unhealthy behaviours, and, thus, supports the need for worldwide effective policy action to control lifestyle-related risk factors for NCDs.

Further research, however, needs to be done in order to develop a more comprehensive measure. The *UBI* suffers from a number of important limitations; it should be considered a first attempt in this research line. Specifically, although data on alcohol consumption include both recorded and unrecorded (i.e., homemade alcohol, illegally produced or sold outside normal government controls) alcohol [17], we do not distinguish between different alcoholic beverages or between moderate consumption and heavy episodic drinking. Similarly, data on cigarette consumption record manufactured and non-manufactured tobacco [18], but we do not consider that the total exposure to tobacco health risks is related to both intensity and prevalence of smoking. Furthermore, a correct measure of eating habits, as noted in the Appendix, has to be based on more dietary characteristics and different nutrient intake besides calorie, total fat and protein consumption. Finally, and perhaps more crucially, working with average country data fails to capture the key role of income inequality in health-related lifestyles within populations.

## Supporting Information

S1 Appendix The nutrition dimension index (*NUTX*), containing Fig A *ECI* and obesity prevalence, and Fig B. *NBD* and obesity prevalence. (DOC)

S1 File Data and results, containing Tables A, B, C, D and E. Table A, Source and short description of each variable. Table B, Raw data (full database, with 159 countries). Table C, Indicators and indices (reduced database, with 112 countries). Table D, Country ranking. Table E, *ECI*, *NBD* and obesity.
(XLS)
