# Supplementary material for: Unhealthy Behaviours: An International Comparison
Source: PLoS One. 2015 Oct 29;10(10):e0141834. doi: 10.1371/journal.pone.0141834 (PMC4626075; doi:10.1371/journal.pone.0141834)
Supplement: S1 Appendix — (DOC) [file pone.0141834.s001.doc]

**S1 Appendix. The nutrition dimension index (*NUTX*).**

At the national level, according to the FAO definitions [19,35] 1) *DES* is the food available for human consumption, expressed in kcal/person/day, and is calculated as the food remaining for human use after deduction of all non-food consumption (exports, animal feed, industrial use, seed and wastage); 2) *ADER* refers to the amount of energy, expressed in kcal/person/day, considered adequate to meet the energy needs for the normative average acceptable weight for the attained height, while performing moderate physical activity in good health; and 3) *AFPS* is the average total fat and protein supply calculated from the national balance food sheets, and expressed in g/person/day (see Table S5).

For each country in the sample, *ECI* is simply *DES* - *ADER*, and *NBD* is calculated as *AFPS* - *RFPI*, where *RFPI* measures the recommended intake of fat and protein, i.e. the correct intake of these nutrients computed by applying to the *ADER* the average optimal share of energy supplied by total fats and proteins – 22.5% and 12.5%, respectively – according to the FAO-WHO [20] population nutrient intake goals. In the case of the UK, for example, *DES* and *ADER* are 3,415 and 2,493 kcal/person/day, respectively. These figures imply an average dietary energy supply adequacy of (3,415/2,493) 137%, and an *ECI* of about (3,415 - 2,493) 922 kcal/person/day. In order to prevent the main NCDs, about 22.5% and 12.5% of the adequate total energy intake should come from fats and proteins, respectively. By applying these recommended values, we find the correct *ADER* composition in terms of fat and protein: (2,493 × 0.225) 560.9 and (2,493 × 0.125) 311.6 kcal/person/day, respectively. Given the average kilocalories per gram of these nutrients (i.e., nine for fat and four for protein), these figures imply an optimal intake of fat and protein of (560.9/9) 62.3 and (311.6/4) 77.9 g/person/day. However, the actual UK average fat and protein supply are 143 and 104 g/person/day, respectively. The result is a diet too rich in total fat and protein (i.e., a non-balanced diet, *NBD*) of about (143 + 104) - (62.3 + 77.9) = 106.8 g/person/day.

The nutrition dimension index (*NUTX*) computed from *ECI* and *NBD* indicators, as described in the main text, is far from being an accurate composite measure of dietary quality. The literature on food pattern quality indicators provides several comprehensive indices of eating habits (such as the Diet Quality Index, the Diet Status Index and the Healthy Eating Index, among others [36]). These indices, however, require more data than those currently available for international country-level comparison. For the purpose of this paper, however, the *NUTX* satisfies two basic conditions: 1) it is compatible with available databases and 2) allows us to summarise two fundamental unhealthy eating habits (that is, ‘an excess of caloric intake’ and ‘a diet too rich in total fat and protein’) that underlie being overweight and obese, one of the main 'intermediate risk factor' for several NCDs. The relationships between *ECI*, *NBD* and the prevalence of obesity are depicted in Figs A and B, respectively (where *OP* indicates the WHO’s [2] age-standardised estimates of obesity prevalence (i.e., BMI ≥ 30) for individuals aged 18+ years (both sex), for each of the 159 countries within the full sample in 2014) (see Table S5). It can be seen from these data that obesity is strictly related to unhealthy habits, as measured by *ECI* and *NBD* (the correlation coefficients between *ECI*, *NBD* and *OP* are 0.54 and 0.58, respectively).

**References**

35. FAO. Integrating food security information in national statistical systems. Experiences, achievements, challenges. Rome: Food and Agriculture Organization of the United Nations; 2014.

36 Drescher LR. Healthy food diversity as a concept of dietary quality. Gottingen: Verlag; 2007.

**Fig A. *ECI* and obesity prevalence*.***

**Fig B. *NBD* and obesity prevalence*.***
